# New global guidelines on sedentary behaviour and health for adults: broadening the behavioural targets

**DOI:** 10.1186/s12966-020-01044-0

**Published:** 2020-11-26

**Authors:** Paddy C. Dempsey, Stuart J. H. Biddle, Matthew P. Buman, Sebastien Chastin, Ulf Ekelund, Christine M. Friedenreich, Peter T. Katzmarzyk, Michael F. Leitzmann, Emmanuel Stamatakis, Hidde P. van der Ploeg, Juana Willumsen, Fiona Bull

**Affiliations:** 1 MRC Epidemiology Unit, Institute of Metabolic Science, University of Cambridge, Cambridge Biomedical Campus, Cambridge, UK; 2 Baker Heart and Diabetes Institute, Melbourne, Australia; 3 Diabetes Research Centre, University of Leicester, Leicester General Hospital, Leicester, UK; 4 Centre for Health Research, University of Southern Queensland, Springfield Central, Australia; 5 College of Health Solutions, Arizona State University, Phoenix, AZ USA; 6 Department of Movement and Sports Sciences, University of Ghent, Ghent, Belgium; 7 School of Health and Life Sciences, Institute for Applied Health Research, Glasgow Caledonian University, Glasgow, UK; 8 Department of Sport Medicine, Norwegian School of Sport Science, Oslo, Norway; 9 Department of Chronic Diseases and Ageing, Norwegian Institute of Public Health, Oslo, Norway; 10 Department of Cancer Epidemiology and Prevention Research, CancerControl Alberta, Alberta Health Services, Calgary, Canada; 11 Departments of Oncology and Community Health Sciences, Cumming School of Medicine, University of Calgary, Calgary, Canada; 12 Pennington Biomedical Research Center, Baton Rouge, LA 70808 USA; 13 Department of Epidemiology and Preventive Medicine, University of Regensburg, Regensburg, Germany; 14 Charles Perkins Centre, School of Health Sciences, Faculty of Medicine and Health, University of Sydney, Sydney, Australia; 15 Department of Public and Occupational Health, Amsterdam Public Health Research Institute, Amsterdam University Medical Centres, Vrije Universiteit Amsterdam, Amsterdam, The Netherlands; 16 Physical Activity Unit, Department of Health Promotion, World Health Organization, Geneva, Switzerland

**Keywords:** Exercise, Physical activity, Sedentary, Guidelines, Public health, Global health, Chronic disease, Cardiovascular, Type 2 diabetes, Cancer, Health promotion

## Abstract

**Background:**

In 2018, the World Health Organisation (WHO) commenced a program of work to update the 2010 Global Recommendations on Physical Activity for Health, for the first-time providing population-based guidelines on sedentary behaviour. This paper briefly summarizes and highlights the scientific evidence behind the new sedentary behaviour guidelines for all adults and discusses its strengths and limitations, including evidence gaps/research needs and potential implications for public health practice.

**Methods:**

An overview of the scope and methods used to update the evidence is provided, along with quality assessment and grading methods for the eligible new systematic reviews. The literature search update was conducted for WHO by an external team and reviewers used the AMSTAR 2 (Assessment of Multiple Systematic Reviews) tool for critical appraisal of the systematic reviews under consideration for inclusion. The Grading of Recommendations Assessment, Development and Evaluation (GRADE) method was used to rate the certainty (i.e. very low to high) of the evidence.

**Results:**

The updated systematic review identified 22 new reviews published from 2017 up to August 2019, 14 of which were incorporated into the final evidence profiles. Overall, there was moderate certainty evidence that higher amounts of sedentary behaviour increase the risk for all-cause, cardiovascular disease (CVD) and cancer mortality, as well as incidence of CVD, cancer, and type 2 diabetes. However, evidence was deemed insufficient at present to set quantified (time-based) recommendations for sedentary time. Moderate certainty evidence also showed that associations between sedentary behaviour and all-cause, CVD and cancer mortality vary by level of moderate-to-vigorous physical activity (MVPA), which underpinned additional guidance around MVPA in the context of high sedentary time. Finally, there was insufficient or low-certainty systematic review evidence on the type or domain of sedentary behaviour, or the frequency and/or duration of bouts or breaks in sedentary behaviour, to make specific recommendations for the health outcomes examined.

**Conclusions:**

The WHO 2020 guidelines are based on the latest evidence on sedentary behaviour and health, along with interactions between sedentary behaviour and MVPA, and support implementing public health programmes and policies aimed at increasing MVPA and limiting sedentary behaviour. Important evidence gaps and research opportunities are identified.

**Supplementary Information:**

The online version contains supplementary material available at 10.1186/s12966-020-01044-0.

## Introduction

Sedentary behaviour is defined as any waking behaviour characterized by an energy expenditure ≤1.5 metabolic equivalents (METs), while in a sitting, reclining, or lying posture [1]. Most desk-based office work, driving or riding in a car, and watching television are examples of sedentary behaviours and can also apply to those unable to stand, such as wheelchair users. In most research studies to date involving ambulatory individuals, sedentary behaviour is typically operationalized as total daily sitting time, television viewing, or low counts on an accelerometer or activity monitor. Sedentary behaviours are considered conceptually distinct from physical inactivity, with the latter referring to performing insufficient amounts of moderate-to-vigorous physical activity (MVPA) to meet current physical activity recommendations. Indeed, it is possible to meet or exceed the public health guidelines for MVPA, and yet also spend most waking hours sedentary. At present, most published population-based estimates of sedentary behaviour are limited to high-income countries, with data on global trends in adults remaining scant. Accelerometer-based estimates from a recent review, derived from large or population-representative studies, indicate that adults spend approximately 8.2 h/day (range 4.9–11.9 h/day) sedentary [2].

Research on sedentary behaviour is relatively recent compared to that of physical activity. Indeed, much of the evidence on the detrimental health effects associated with sedentary behaviour has rapidly accumulated within the past decade. However, there have been notable developments, and the evidence-base is now at a level where dose-response relationships between sedentary behaviour and multiple health outcomes are being systematically examined, along with the interplay between sedentary behaviour and MVPA [3–7]. Given its high prevalence and increasing concern of the potential impact on public health, a growing number of countries are interested in and developing recommendations on sedentary behaviour at varying levels of specificity, either by incorporating them into their physical activity guidelines or by issuing specific sedentary behaviour guidelines (e.g. [8–12]).

In 2018, the World Health Organisation (WHO) was requested to update the *2010 Global Recommendations on Physical Activity for Health* based on the latest available science, including sedentary behaviour, as part of global efforts to support countries to implement recommendations set out in the *Global Action Plan on Physical Activity 2018–2030* and achieve a 15% reduction in physical inactivity by 2030 [13]. WHO commenced this program of work and convened an international group of public health scientists and practitioners to serve on the Guideline Development Group (GDG) [14]. The purpose of this paper is to briefly summarize and highlight the scientific evidence that underpinned the new sedentary behaviour guidelines and its strengths and limitations. We also discuss the public health importance and practical implications of these new guidelines and outline several important evidence gaps and future research directions.

## Methods

The guideline development process followed WHO protocols [15] and included establishing a guideline development group (GDG) who met in July 2019 to review and finalise the scope and agree on the methods. Full details of the procedures for identifying and grading the evidence are described in detail elsewhere [14, 16]. The evidence-base around sedentary behaviour and health outcomes in youth are detailed separately [17].

Briefly, the critical outcomes that were examined and the set of PI/ECO (Population, Intervention/Exposure, Comparison, Outcome) questions are shown in Table 1. Literature searches were undertaken to update the most recent and relevant systematic reviews for the critical outcomes only and not the important outcomes (see Table 1), which for sedentary behaviours and adults were identified to be the comprehensive syntheses of evidence undertaken by the *2018 Physical Activity Guidelines Advisory Committee (PAGAC) Scientific Report* from the United States [9].

**Table 1:**
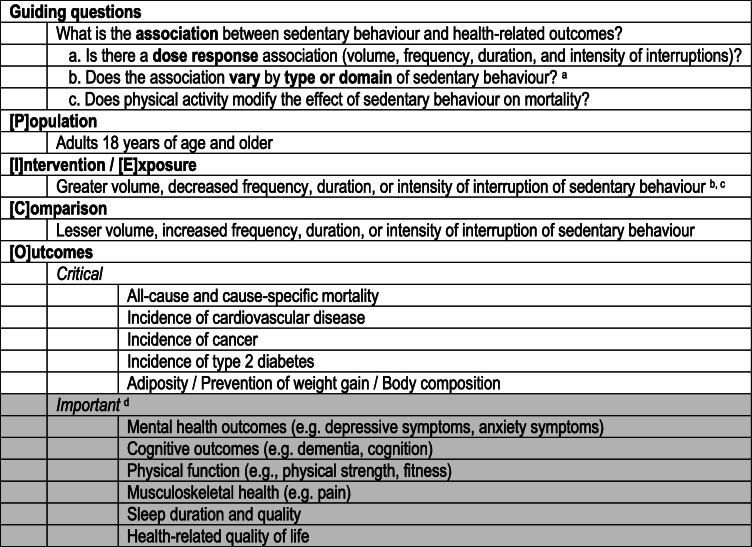
Scope and PI/ECO questions related to sedentary behaviour and health outcomes in adults

^a^ The search to update the evidence used the same search terms as PAGAC and they were likely broad enough to pick up any relevant systematic reviews on type or domain of sedentary behaviour. However, it is noted that PAGAC did not specifically address this question in their final scope and thus some evidence may have been missed

^b^ Sedentary behaviour exposure measures operationalised as either: total sitting time, screen-time, leisure-time sitting, occupational sitting time, accelerometer measured sedentary time [1, 9]

^c^ Evidence on bouts and breaks in sedentary behaviour was also examined. A ‘bout’ of sedentary behaviour can be operationalized as a period of uninterrupted sedentary time, whereas a ‘break’ in sedentary behaviour can be operationalized as a non-sedentary bout in between two sedentary bouts [1]

^d^ New searches were not conducted for the ‘important’ outcomes due to anticipated time/resource constraints, hence no results nor conclusions are reported for these outcomes

The literature search update was conducted for WHO by an external team and reviewers used the AMSTAR 2 (Assessment of Multiple Systematic Reviews) tool for critical appraisal of the systematic reviews under consideration for inclusion [18]. The Grading of Recommendations Assessment, Development and Evaluation (GRADE) method was used to rate the certainty (i.e. very low to high) of the evidence for each PI/ECO (see Additional File 1) [19, 20].

The GDG considered both the evidence reported by the PAGAC and new reviews identified to make recommendations on health outcomes and sedentary behaviour for all adults and older adults. Other considerations such as values, preferences and risks and identified evidence gaps were also appraised. Observational evidence from reviews including more well-conducted longitudinal studies were upgraded to better reflect the increased certainty in findings and improved inferences about the causal structure of associations between sedentary behaviour and health outcomes from such studies. Greater emphasis was given to evidence provided by reviews graded moderate or above, and to those reviews providing evidence from studies using measures of total sedentary or sitting time, or device-based measures of sedentary time, where available.

## Results

The PAGAC report provided systematic-review-level evidence published from 2011 to 2016 on sedentary behaviour and all-cause (*n* = 9), cardiovascular disease (CVD; *n* = 5) and cancer (*n* = 5) mortality, and type 2 diabetes (*n* = 5), weight status (*n* = 2), CVD (*n* = 5) and cancer (*n* = 8) incidence in adults. PAGAC applied a modified evidence grading protocol which is fully described elsewhere [9, 21, 22].

The updated search for systematic reviews identified 22 potential new reviews published from 2017 to August 2019. Of these, 17 reviews met inclusion criteria (five were excluded because their study exposures or design were out-of-scope) and a further three reviews were excluded because they had critically low credibility ratings (Table 2). The 13 remaining reviews provided updated evidence on all-cause, CVD and cancer mortality, type 2 diabetes, CVD and cancer incidence, and adiposity. For a detailed summary of the grading of meta-analyses and systematic reviews that contributed new evidence to inform the GDG conclusions, see the WHO report [16] and Web Annex Evidence Profiles (Tables B.2.a-e).

**Table 2:** Credibility ratings for identified new reviews according to 16 item AMSTAR 2 tool

Author, Year	ACM	Cause-specific mortality	CVD	Cancer	Diabetes	Adiposity	Last Search Date	AMSTAR 2 ^d^
Ahmad 2017 [23]			X		X	X	Dec 2016	Moderate
Bailey 2019 [24]			X		X		Feb 2019	Moderate
Berger 2019 [25]		Prostate		Prostate			Jan 2019	Moderate
Chan 2019 [26]				Breast			Apr 2017	Moderate
del Pozo-Cruz 2018 [6]	X		X		X	X	Dec 2016	Moderate
Ekelund 2018 [3] ^a^		CVD, Cancer					Oct 2015	Moderate
Ekelund 2019 [5]	X						Jul 2018	Moderate
Ku 2018 [27]	X						Jan 2018	Moderate
Ku 2019 [28]	X						Mar 2019	Moderate
Lee 2019 [29]				Ovarian			Dec 2017	Critically Low ^e^
Ma 2018 [30]				Colorectal			Feb 2017	Critically Low ^e^
Mahmood 2017 [31]				Colorectal			Dec 2015	Low
Mañas 2017 [32]	X ^b^						Oct 2016	Critically Low^e^
Patterson 2018 [7]	X	CVD, Cancer			X		Sep 2016	Low
Shepard 2017 [33]				Bladder			Jun 2016	Critically Low^e^
Wang 2018 [34]				Colorectal			Sep 2018	High
Xu 2019 [35] ^c^	X						May 2018	Low

^a^ Secondary data analysis of 2016 review [4]

^b^ Not included for all-cause mortality given better quality reviews reporting this outcome

^c^ Individual participant data meta-analysis

^d^ See WHO report [16] for details on the AMSTAR 2 tool to rate the credibility of the evidence and the full evidence profiles

^e^ Reviews rated as having critically low credibility were not incorporated into the final evidence profiles

### Evidence

#### All-cause and cause-specific mortality (Web Annex, Table B.2.a)

A recent high certainty harmonised meta-analysis (eight prospective studies; *n* = 36,383) in middle aged and older adults (mean age 62.6 years; 72.8% women) showed a non-linear positive dose-response relationship between accelerometer-measured sedentary time and *all-cause mortality* [HRs and 95% CI per quartile of higher sedentary time relative to the least sedentary referent, after adjustment for potential confounders including time spent in MVPA: 1.28 (1.09 to 1.51), 1.71 (1.36 to 2.15), and 2.63 (1.94 to 3.56)], with mortality risks increasing gradually from approximately 7.5 to 9 h/day and becoming more pronounced from > 9.5 h/day [5]. Another recent comprehensive dose-response meta-analysis of over a million participants [7] also found non-linear positive associations for total sedentary behaviour (mostly self-reported) with *all-cause mortality* (RR per 1 h/day = 1.01 (95% CI = 1.00–1.01) for ≤8 h/day and 1.04 (95% CI = 1.03–1.05) for > 8 h/day of exposure) and *cardiovascular disease mortality* (RR = 1.01 (95% CI = 0.99–1.02) for ≤6 h/day and RR = 1.04 (95% CI = 1.03–1.04) for > 6 h/day), although associations with *cancer mortality* were not statistically significant, after adjustment for physical activity.

A new harmonized meta-analysis (CVD mortality, 9 studies, *n* = 850,060; Cancer mortality, 8 studies, *n* = 777,696) provided high certainty evidence on whether associations between self-reported sitting (and TV viewing) varied among different strata of MVPA for CVD and cancer mortality [3]. Significant dose–response associations (9–32% higher risk) were shown for sitting time and *CVD mortality* in the inactive, lowest quartile of MVPA (~ 5 min/day). More specifically, the hazard of cardiovascular disease mortality was 32% higher in those who sat > 8 h/day compared with the reference group (< 4 h/day). The results were less pronounced but remained statistically significant compared with the reference group for the second (HR = 1.11, 95% CI = 1.03–1.20) and third quartiles (HR = 1.14, 95% CI = 1.03–1.26) of MVPA, but this association was mitigated in the most active quartile (~ 60–75 min/day). This review also found that associations for sedentary behaviour and *cancer mortality* were generally weaker, although a 6–21% higher dose-related risk was observed with higher sitting time (particularly > 8 h/day) among those who were in the lowest quartile of MVPA (~ 5 min/day), with hazard ratio’s largely attenuated in the highest quartile of MVPA. These new findings build upon previous harmonized meta-analyses [4] and show overall that the associations of sedentary behaviour and risk for all-cause, CVD and cancer mortality appear to be more pronounced at lower levels of MVPA than at higher levels, but that higher levels of MVPA (i.e. about 60–75 min/day) can largely mitigate the increased risks of sedentary behaviour.

#### Type 2 diabetes, CVD, and cancer incidence (Web Annex, Table B.2.b-d)

Two new moderate certainty reviews [including eleven prospective studies (*n* = 400,292) and five prospective studies (*n* = 4575) with two duplicates, and slightly different foci in terms of exposure and study inclusion criteria], examined associations of total daily sitting time [24] and total sedentary behaviour (mostly self-reported) [7] with type 2 diabetes incidence. Small linear associations were observed for increments of 1 h/d in total sedentary behaviour (RR = 1.01, 95% CI = 1.00, 1.01) [7], while higher levels of total sitting time were also associated with increased risk of diabetes incidence (HR = 1.11, 95% CI = 1.01, 1.19) after adjustment for physical activity [24]. Bailey et al. also found an increase in incident CVD risk (HR = 1.29 (95% CI, 1.27 to 1.30) with total sitting, which was attenuated following statistical adjustment for physical activity (HR = 1.14 (95% CI, 1.04 to 1.23)) [24]. Four new reviews [25, 26, 31, 36] reporting on sedentary behaviour and cancer incidence all had very low to low certainty ratings, mostly attributable to a lack of adjustment for confounding variables, indirectness in terms of diversity of outcome assessment, and high statistical heterogeneity. However, evidence from three meta-analyses [37–39] previously summarized [9] provided moderate certainty evidence for an association between sedentary behaviour and type cancer incidence (endometrial, colon, and lung cancers).

#### Adiposity (Web Annex, Table B.2.e)

Only two new reviews for adiposity [6, 40] were identified. Both were of very low certainty and included mostly cross-sectional studies with higher risk of bias [i.e. thirteen cross sectional studies and one prospective study (*n* = 13,395) and six cross-sectional studies (*n* = 4774)]. The results reported were consistent with previous reviews [9] and showed limited, heterogeneous, and/or low certainty evidence for a small association between sedentary behaviour and adiposity markers (i.e. BMI and waist circumference), and low/insufficient certainty evidence for a dose-response relationship.

### Overall conclusions, extrapolation to sub-populations, and WHO guidelines

Table 3 provides a summary of the relationships and level of evidence for each health outcome examined with sedentary behaviour, which was largely consistent and complementary between the systematic reviews. Overall, there was moderate certainty systematic review evidence for a ***direct association*** (i.e. an ‘independent’ association after adjustment for potential confounders, including MVPA) between higher amounts of sedentary behaviour and increased risk of all-cause, CVD and cancer mortality, as well as CVD, cancer, and type 2 diabetes incidence; however, evidence was limited and of low certainty for adiposity markers. Moderate certainty evidence also supported a non-linear ***dose-response relationship*** between sedentary behaviour and all-cause, CVD and cancer mortality, and incident CVD. This evidence provided sufficient support for new recommendations to limit sedentary time and replace it with activity of any intensity to reduce health risks (see Table 4) and the benefits of limiting sedentary behaviour were deemed to outweigh the risks. However, given the considerable variations in how sedentary behaviour was assessed (via self-reported sitting time, television viewing time, or device-based (accelerometer) assessments) and reported in studies, it was concluded there was insufficient evidence to set ***quantified (time-based)*** recommendations (Table 3). It was also considered probable that specific thresholds for sedentary time were likely to vary across health outcomes, by levels of MVPA (see below), and among population sub-groups.

**Table 3:** Summary of relationships and level of evidence for sedentary behaviour and each health outcome in adults

Health Outcomes	Evidence for association	Evidence for dose-response ^c^	Evidence for variation in association by physical activity	Evidence for type or domain of sedentary behaviour
All-cause mortality	Moderate	Moderate	Moderate	Insufficient
CVD mortality	Moderate	Moderate	Moderate	Insufficient
Cancer mortality	Moderate	Moderate	Moderate	Insufficient
Incident type 2 diabetes	Moderate	Low	Insufficient	Insufficient
Incident CVD	Moderate	Moderate	Insufficient	Insufficient
Incident cancer ^a^	Low-moderate ^b^	Low	Insufficient	Insufficient
Adiposity	Low/insufficient	Low/insufficient	Insufficient	Insufficient

See the WHO report [16] for details on the framework to rate the certainty of the evidence and the full evidence profiles

^a^ Includes endometrial, colon, and lung cancers. Evidence graded very low to low certainty for other cancer types

^b^ Level of evidence rating based on evidence from PAGAC reviews [9]

^c^ It was concluded that there was insufficient evidence to set quantified (time-based) recommendations

**Table 4:** The new WHO sedentary behaviour guidelines for adults and older adults^a^ (strong recommendation, moderate certainty evidence)

1. Adults and older adults should limit the amount of time spent being sedentary. Replacing sedentary time with physical activity of any intensity (including light intensity) has health benefits.	
2. To help reduce the detrimental effects of high levels of sedentary behaviour on health, and older adults should aim to do more than the recommended levels of moderate-to-vigorous physical activity.	

^a^ Guidelines 1 and 2 were extrapolated to those living chronic conditions and/or disabilities, and only guideline 1 was extrapolated for pregnant and postpartum women (strong recommendation, low certainty evidence; without contraindication)

There was moderate certainty evidence that the associations between sedentary behaviour and all-cause, CVD and cancer mortality ***vary by level of MVPA*** when modelled in joint or stratified analyses. In other words, higher amounts of MVPA (i.e. about 60–75 min/day) can attenuate the detrimental association between sedentary behaviour and health outcomes. This underpinned additional guidance around increased levels of MVPA in the context of high levels of sedentary time to help reduce the risks (see Table 4).

In addition to overall volume of sedentary behaviour, the patterns by which sedentary behaviour is accrued were reviewed. However, consistent with previously summarized evidence syntheses [9], there was insufficient or low-certainty systematic review evidence to make recommendations on the frequency and/or duration of ***bouts or breaks*** in sedentary behaviour. The possibility that some ***types or domains*** of sedentary behaviour may be more detrimental to health than others, both in terms of their direct associations and their potential to displace time spent in more healthful physical activity, was also considered. For example, some studies report stronger associations with sedentary behaviour reported as TV viewing compared with total sitting time [7]. This may be due to differences in the behaviours themselves, differential measurement error/validity, or differences in residual/unmeasured confounding associated with the self-report measures and instruments, but further research is still needed to untangle this. Similarly, some misclassification may occur from device-based measures of sedentary time as many of these device placements (e.g. wrist, waist) do not currently distinguish between positions (e.g. lying, sitting, and standing still). At present, there is insufficient/low certainty review-level evidence directly comparing associations between different types/domains of sedentary behaviour to make any conclusions or recommendations.

Direct evidence on sedentary behaviours and health outcomes in people living with ***disabilities*** or ***chronic conditions,*** or ***pregnant or postpartum women,*** remains sparse. However, the evidence reviewed in the general adult population, including the benefit of undertaking more MVPA to help counteract the potential risks of high levels of sedentary behaviour, was considered applicable (assuming no contraindications) and therefore extrapolated to inform guidelines on sedentary behaviour for these specified sub-populations [41, 42] for a common set of critical health outcomes, with a downgrading of certainty to reflect indirectness (see Table 4). In extrapolating this evidence to people living with disabilities, it was recognized that certain population groups, such as wheelchair users, unavoidably sit for long periods of time and sitting may therefore be the norm. For these groups, sedentary behaviour is best defined based on the low energy expenditure component, rather than the postural component (e.g. moving in a power chair or being pushed in a wheelchair). The final guidelines on sedentary behaviour for all adults and older adults are detailed in Table 4.

## Discussion

In the past decade, there has been a rapid accumulation of evidence on the prospective associations between sedentary behaviour and several critical public health outcomes (see Table 3). With increasing concerns around low prevalence of physical activity and rising levels of sedentary behaviour among many populations worldwide, important public health gains could be made by limiting excessive sedentary behaviour and replacing it with more physical activity of light, moderate or vigorous intensity. As such, the incorporation of new evidence-based recommendations on sedentary behaviour for all adults and older adults within the 2020 WHO guidelines marks an important step forward, while complementing and extending existing physical activity guidelines.

Although the evidence reviewed shows that sedentary behaviour is clearly related to several health outcomes, there remains some imprecision and uncertainty in the characteristics of the specific dose-response curves, which in turn has made it difficult to provide specific quantitative public health recommendations. As researchers and policy makers enter this new era of joint physical activity and sedentary behaviour recommendations, three important evidence and practice gaps warrant further contextualisation and discussion:

***1. No specific or quantified (time-based) threshold for sedentary time:*** Identifying whether there is a threshold of sedentary behaviour which is associated with increased health risk is of public health and policy relevance. Although there was some consistency in the positive dose-response relationships between sedentary time and mortality outcomes (but less so for type 2 diabetes and cancer incidence or adiposity) to support clear statements to limit sedentary time overall (see Table 3), there was insufficient evidence to identify a specific time-based threshold. Limitations in the evidence included the variations in how sedentary time has been measured or reported across all the major reviews, which complicates the identification of specific quantitative thresholds. Moreover, the evidence reviewed showed that thresholds of increased risks for sedentary time are likely to vary depending upon the level of MVPA (as described in the second sedentary behaviour recommendation; also see *point 2*), by health outcome, and among different population sub-groups (e.g. defined by age, sex, race/ethnicity, socioeconomic status, or weight status, etc). Future reviews that include well-harmonised and/or device-based measures of total sedentary time in ethnically and culturally diverse populations could help inform more specific quantifications around the amounts of sedentary time that significantly increase health risks. However, evidence to inform such sedentary guidance is complex and will also need to be considered within the context of its inter-relationships with physical activity of various types/intensities.

**Fig. 1:**
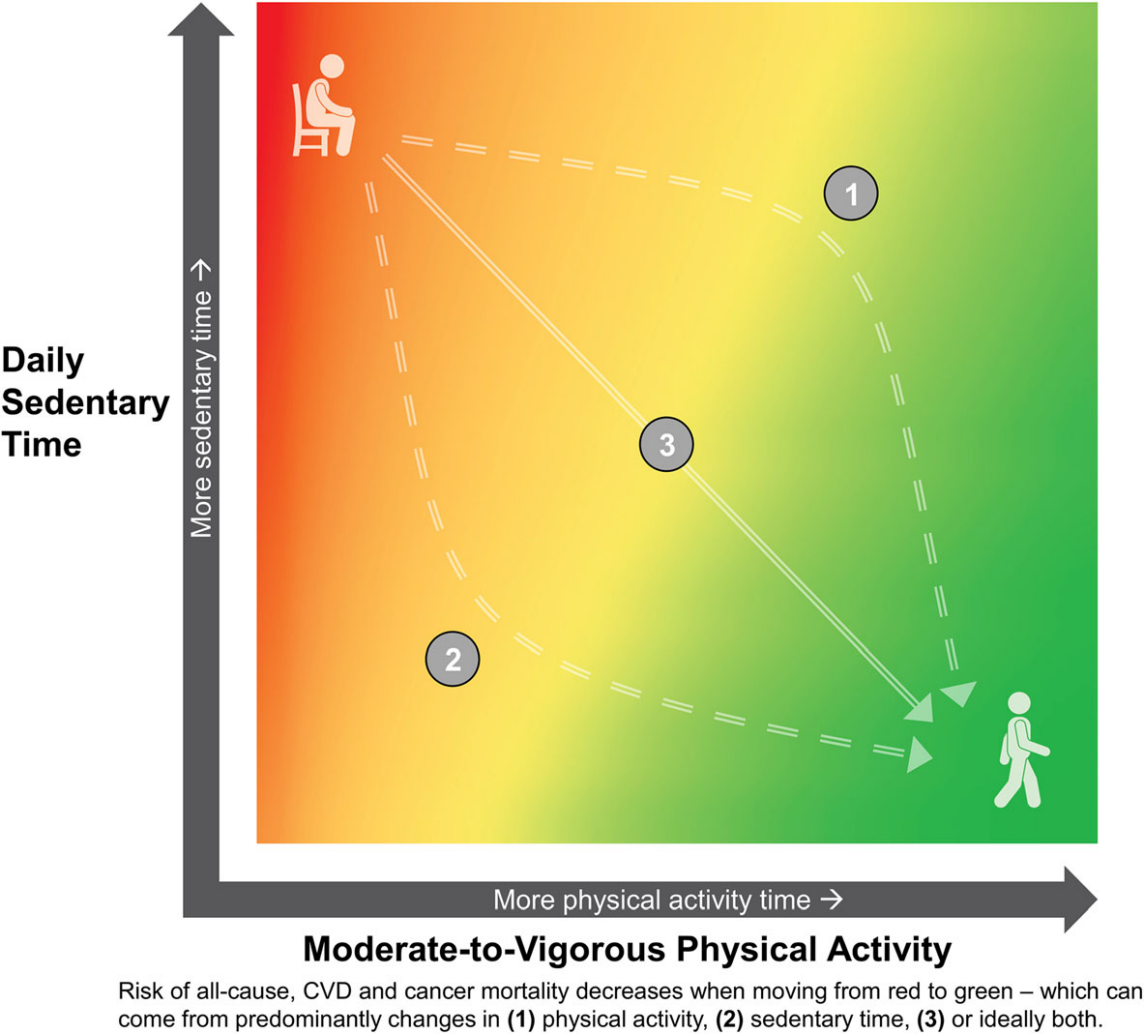
Joint associations of sedentary (sitting) time and MVPA with risk of all-cause mortality based on data by Ekelund et al. [4] – now also broadly applicable for risk of CVD and cancer mortality [3]. Orange and yellow shading represents transitional decreases in risk. For context, data analysis ranges for all-cause mortality [4] were based on four levels of self-reported sedentary time (< 4, 4–6, 6–8, > 8 h/day) and MVPA (∼5, 25–35, 50–65, 60–75 min/day), but specific scales are intentionally left blank and could vary considerably for either device-based measures (e.g. hip or thigh accelerometry), by different health outcomes (e.g. type 2 diabetes, adiposity), or by different sub-populations (e.g. frail/elderly adults, people living with some chronic conditions or disabilities). Heat map adapted from the *PAGAC* [9] report

***2. The feasibility of achieving or exceeding the upper limits of MVPA guidelines to reduce the detrimental effects of “high levels” of sedentary behaviour:*** The new evidence reviewed demonstrating that high levels of MVPA (i.e. > 300 min/week) can largely offset the mortality risks associated with sedentary behaviour is good news for those who are highly sedentary, but who are also able to achieve high levels of MVPA. This evidence emphasizes the role of MVPA in offsetting the potential harms associated with excessive sedentary time (see Table 4). However, achieving such high levels of MVPA may be a challenge for large segments of the population, as illustrated by the high proportion of people not meeting the lower limit for MVPA guidelines [43, 44]. Dual sedentary behaviour guidelines therefore emphasize and support added *flexibility* in options, through encouraging the use of *multiple approaches* or *strategies* to reduce risk. These could include lowering total sedentary time (and likely increasing light-intensity activity), increasing MVPA time or, ideally, some combination of both strategies. This integration concept is illustrated elegantly by the heat map developed by the *PAGAC* [9], which shows conceptually that many combinations of less sedentary time and more MVPA can be associated with a reduced risk of all-cause mortality (see Fig. 1) – evidence which has now been extended to include CVD and cancer mortality outcomes [3]. Importantly, the new WHO guidelines point to the importance of attending to *both* physical activity (i.e. of light, moderate, and vigorous intensity) and sedentary time to try to optimize the “balance” of these behaviours for better health. The dual sedentary recommendations also promote more *inclusivity* towards a broader variety of sub-populations – including large portions of the population who are physically inactive or obese, or who have chronic conditions or disabilities – for whom achieving high levels of MVPA may be challenging.

***3. No specific recommendations on how to break up sedentary behaviour:*** Information on sedentary break and bout accumulation patterns in relation to health outcomes represents a promising and potentially powerful public health messaging tool. However, operationalising and analysing sedentary break and bout accumulation pattern data, distinct from the volume of time spent in sedentary and active behaviours, requires more clarity and detailed interrogation. There was insufficient or low-certainty systematic review evidence on the frequency and/or duration of bouts or breaks in sedentary behaviour with health outcomes to make specific recommendations. A lack of evidence to date is likely due in part to the reliance on device-based measures, and some heterogeneity/limitations in how bouts and breaks in sedentary time are summarized. Some emerging but limited evidence is available from short to medium duration randomized controlled trials (RCTs) and prospective cohort studies with clinical endpoints. However, it should also be noted that most of the review-level evidence is based on cross-sectional [45] or acute laboratory-based [46] studies, or free-living short to medium duration intervention studies with behavioural or surrogate disease risk biomarkers as primary outcomes [47–49].

### Key limitations/gaps in the evidence base and future directions

Several limitations and gaps in the current evidence base were identified and future research recommendations generated from this work, some of which are also broadly covered in a separate paper [50]. Key future directions and opportunities are detailed below. More research in all of these areas would lead to more specificity and generalisability in guidelines, such as what types/domains of sedentary behaviours to limit most, the role of standing in replacing sitting, which types/domains of sedentary behaviours may be neutral or even health-promoting, and specific daily or weekly time-based thresholds/ranges above which there are important health risks. Advances in measurement tools and analytics, as well as better data harmonization or pooling across studies, should support and provide important insights towards these areas. However, it is important that such research is paralleled by greater efforts to improve global surveillance and data collection across a diversity of sub-populations and low- and middle-income countries (LMICs), including those from more disadvantaged/vulnerable backgrounds, where evidence remains particularly scant.

***1. Measurement and analytics:*** Most of the evidence reviewed by the GDG was derived from self-reported sitting and TV viewing time, with less evidence from device-based measures of sedentary behaviour or sitting per se. There is a need to develop and incorporate better field-based measurement methods to adequately quantify time spent in sedentary behaviours. Such methods should quantify both postural and energy expenditure components, consistent with the accepted sedentary behaviour definition, and ideally also capture more detailed information on the type and domain of sedentary behaviour (e.g. occupational, sedentary screen time, active/passive sedentary behaviours, upper and lower-body fidgeting, etc). To capture all such elements will likely require combinations of both self-report and device-based methods, including possible use of wearable cameras. Moreover, since sedentary time, light-intensity physical activity, MVPA and sleep all co-exist and interact within a finite 24-h day or energy total, analytical methods that better account for the relative time- and energy-use compositions of these behaviours will provide more useful insights into correlates and behavioural associations with health outcomes [51–53]. Similarly, better analytical methods to identify, standardize, and summarize information on sedentary breaks and bout accumulation patterns (distinct from total sedentary volume or other time-uses) will provide more useful information when examining associations with prospective health outcomes.

***2. Outcomes, mechanisms, and context:*** More prospective evidence is needed on a broader range of health and psycho-biological outcomes. The bulk of the literature so far, including the evidence synthesis for this review, relies on mortality or cardiometabolic outcomes. Similar to physical activity, future guidelines should ideally be based on evidence from a more comprehensive suite of important health or health-related outcomes, including: specific cancers; mental health (including affective responses); cognitive/brain health; musculoskeletal health and falls; social outcomes; and quality of life. More experimental and etiological research that informs biological mechanisms (both acute and longer term) and potential causal pathways linking sedentary behaviour with health outcomes will also be highly informative [54]. Examples include: experimental evidence on direct effects of exposures to different types, postures, or patterns of sedentary behaviour; residual/unmeasured confounding issues (e.g. socioeconomic status, diet, occupation type, mental health, functional status, cancer screening); untangling issues around reverse or bi-directional causality and mediation (e.g. for type 2 diabetes and adiposity); effect modification by key demographic and personal characteristics (e.g. sex, age, race/ethnicity, chronic conditions, disabilities, socioeconomic status, occupation type, adiposity, and cardiorespiratory fitness); and interactions between sedentary time and physical activity across the intensity spectrum (e.g. light, moderate, and vigorous).

***3. Examining options for limiting sedentary behaviour in RCTs:*** Building on previous points, research that encapsulates the full 24-h range of behaviours (e.g. sleep, sedentary time, light-intensity physical activity, and MVPA) will inform sedentary behaviour guidelines by providing guidance on how to replace sedentary time optimally. In particular, the positive or negative consequences of replacing sedentary time with additional sleep duration, standing, and various other activities within the wide range of light-intensity activities, and for whom, is of interest. Ideally, these questions should be examined within the context of high-quality RCTs targeting a variety of sub-populations, using harmonizable measures of these behaviours. This research will allow specific replacement effects to be more reliably ascertained through later individual-level participant data analyses and meta-analytic approaches [47]. Finally, a better understanding of the key biological or modifiable determinants (or “drivers”) of sedentary behaviour (a behaviour which can often be habitual and socially/environmentally reinforced) will also be crucial in informing the design, targeting, and implementation of the above RCTs, as well as more effective, evidence-based interventions and policies to change sedentary time [55].

***4. An uneven evidence base generated in high-income countries:*** To date, most evidence on sedentary behaviour (and physical activity) has been built on studies examining populations predominately from western high-income countries. Therefore, the prevalence, context, and generalisability of different amounts, types, and patterns of sedentary behaviour – particularly in terms of determinants, health impacts, and targeted interventions – remains less clear for populations in LMICs. A global perspective and more high-quality empirical data in a diversity of sub-populations and LMICs is therefore needed. This need is particularly pressing since LMICs are already experiencing economic and societal transitions that are expected to lead to rapid urbanisation and more sedentary jobs/societies. Indeed, populations in LMICs are already experiencing higher global disease burden or differential disease patterns compared to high-income countries [56, 57]. It seems likely that some underlying institutional, social, and cultural practices between countries will mean that sedentary and active living are viewed or influenced in different ways [58], such as leisure or occupational sedentary time representing higher social status or active transport representing poverty. These unique determinants/macrolevel drivers and potential differential health impacts of sedentary behaviour require more detailed data and understanding across a wider range of countries, with a focus on disadvantaged populations and LMICs. Recent global surveillance data, though somewhat limited, indicates that self-reported sedentary time varies substantially between high- and low-income countries [59], with high-income countries reported sedentary time almost double that of low income countries (4.9 vs 2.7 h/day). Moreover, the contextual patterns in which sedentary behaviours occur also vary by indices of socioeconomic status and markers of social disadvantage. For example, in high-income countries, occupational sedentary time tends to be higher among those with higher educational attainment or income, whereas TV viewing levels are often higher among those in lower socioeconomic positions [60]. Understanding and addressing potential social and cultural inequities in the determinants, prevalence, and health impacts of different sedentary behaviours, and appropriate opportunities and strategies to intervene, is therefore an important area in need of more research.

### Policy and practice implications and opportunities

From a practical perspective, policy makers should view the new WHO sedentary behaviour guidelines as complementary and reinforcing to physical activity guidelines and public health endeavours to reduce the risk of non-communicable diseases. The benefits of MVPA are well-established, and these new sedentary recommendations show that substantial health gains also exist from promoting all adults to limit high levels of sedentary time, and to replace sedentary behaviours with physical activity of *any* intensity. These recommendation support new opportunities for more comprehensive messages and policies to help improve health, such as “move more” *and* “sit less” (or limit sedentary behaviour in non-ambulatory individuals), and even without specific thresholds, such messages are intrinsically synergistic from a public health perspective.

These new sedentary behaviour recommendations should be disseminated to key audiences and across multiple settings, broadening the potential options for health promotion and non-communicable disease prevention/management initiatives. Indeed, as emphasized in the *Global Action Plan on Physical Activity 2018–2030* [13], there are multiple ways to be more active, multiple policy choices, settings and opportunities, and multiple benefits. The same is true of sedentary behaviour; thus, a *combined* emphasis on policies and initiatives to change both physical activity and sedentary behaviours is needed to help move the physical activity agenda forward, and importantly scale-up more action at the national and global level.

The synergies between physical activity and sedentary behaviour calls for both interdisciplinary and intersectoral population health action. However, this should be supported by and will require a deeper understanding of the complexity of sedentary behaviour (e.g. from measurement/operationalization, to determinants, and health impacts) and its inter-relationships with other behaviours under different contexts. These inherent complexities suggest we should be working with different fields, expertise, and constituencies beyond public health – such as urban planners, employers, educators, and the sport sector – to create more “activity-friendly” communities and social/built environments. A solutions-oriented approach, as well as a focus on different behavioural settings (e.g. neighbourhoods, schools, domestic/homes, workplaces, and transport/commuting) and creating stronger partnerships with communities, healthcare, employers, businesses, and government, transport and industry sectors, should also be emphasised [55, 61]. Most importantly, public health efforts will need to move beyond short-term implementation and impact, and more towards achieving system embeddedness, co-benefits, and translation at scale into policy and practice [62, 63]. Moreover, the appropriateness and efficacy of all such approaches and policies will need to be tested in a diversity of populations – including those from disadvantaged backgrounds or LMICs, those living with chronic diseases or disabilities, and across different cultural backgrounds.

## Conclusion

The *WHO 2020 guidelines on physical activity and sedentary behaviour* provide new guidance on sedentary behaviour and its interrelationships with physical activity. They provide a broader, mutually reinforcing set of behavioural targets to help improve population health. Identified evidence gaps underscore the need for further research, especially among certain sub-populations and in diverse contexts including more LMICs. An important challenge will now be to identify effective, sustainable, and scalable approaches for limiting sedentary behaviour and increasing physical activity among those who need it most, particularly more vulnerable populations. These new sedentary behaviour guidelines should provoke more targeted research in this area and be a catalyst for more system-wide policies, programs, and initiatives to help improve global health.

## Supplementary Information

**Additional file 1.** Grading the body and certainty of evidence.

## Data Availability

Not applicable.
