# Supplementary material for: New global guidelines on sedentary behaviour and health for adults: broadening the behavioural targets
Source: Int J Behav Nutr Phys Act. 2020 Nov 26;17:151. doi: 10.1186/s12966-020-01044-0 (PMC7691115; doi:10.1186/s12966-020-01044-0)
Supplement: Supplementary file 1 — Additional file 1. Grading the body and certainty of evidence. [file 12966_2020_1044_MOESM1_ESM.docx]

**Additional File 1:** Grading the body and certainty of evidence

The Grading of Recommendations Assessment, Development and Evaluation (GRADE) method was used to rate the certainty of the evidence for each PI/ECO (1), based on the underlying evidence in the reviews. When available, the GRADE “Evidence Profiles” or “Summary of Findings” tables from each review, were used as a starting point. If no table was available within the existing systematic reviews, Evidence Profile tables for each population and outcome of interest were constructed.

The following GRADE criteria were considered: study design, risk of bias, consistency of effect, indirectness, precision of effect, and other limitations, including publication bias and factors for upgrading observational evidence (magnitude of effect, dose-response, and effects of confounders). Observational evidence from well-conducted longitudinal studies within the reviews was also upgraded to better reflect the increased certainty in findings regarding associations between sedentary behaviour and outcomes from such studies more appropriately. Studies that evaluated intermediate/indirect outcomes were not necessarily downgraded if prioritized by the Guideline Development Committee; the GRADE rating reflects the certainty in effects on those outcomes. In some cases, the GRADE ratings from existing reviews were modified to ensure consistency in application of GRADE methods. The certainty in the body of evidence for each outcome was assigned based on the following guidance (2):

| **High** | We are very confident that the true effect lies close to that of the estimate of the effect |
| --- | --- |
| **Moderate** | We are moderately confident in the effect estimate: The true effect is likely to be close to the estimate of the effect, but there is a possibility that it is substantially different |
| **Low** | Our confidence in the effect estimate is limited: The true effect may be substantially different from the estimate of the effect |
| **Very low** | We have very little confidence in the effect estimate: The true effect is likely to be substantially different from the estimate of effect. |

Further details on the GRADE methods and process of going from evidence to recommendations (i.e. GRADE Evidence to Decisions (EtD) framework) can be found in the main WHO report (3). GRADE tables detailing the overall certainty of evidence, taking into consideration the risk of bias, inconsistency, imprecision, indirectness of the evidence and publication bias across each outcome for each PI/ECO are also available in Web Annex Evidence Profiles (Tables B.2.a-e) of the WHO report (3).

1. Guyatt GH, Oxman AD, Vist GE, Kunz R, Falck-Ytter Y, Alonso-Coello P, et al. GRADE: an emerging consensus on rating quality of evidence and strength of recommendations. BMJ. 2008;336(7650):924-6.

2. Balshem H, Helfand M, Schunemann HJ, Oxman AD, Kunz R, Brozek J, et al. GRADE guidelines: 3. Rating the quality of evidence. Journal of clinical epidemiology. 2011;64(4):401-6.

3. Bull F, Willumsen J. World Health Organization. Guidelines on physical activity and sedentary behaviour. World Health Organization: Geneva, Switzerland; 2020.
